# Volatile Organic Compounds, Evaluation Methods and Processing Properties for Cooked Rice Flavor

**DOI:** 10.1186/s12284-022-00602-3

**Published:** 2022-10-29

**Authors:** Zichen Zheng, Chao Zhang, Kewei Liu, Qiaoquan Liu

**Affiliations:** 1 College of Mechanical Engineering, Yangzhou University, 196 West Huayang Road, Yangzhou, 225127 Jiangsu Province People’s Republic of China; 2 Key Laboratory of Crop Genetics and Physiology of Jiangsu Province, Co-Innovation Center for Modern Production Technology of Grain Crops of Jiangsu, College of Agriculture, Yangzhou University, Yangzhou, 225009 People’s Republic of China

**Keywords:** Cooked rice, VOCs, Evaluation methods, Processing properties analysis

## Abstract

Rice (*Oryza sativa* L.), as the main refined grain in China, has attracted much attention in terms of quality. Rice is usually consumed after cooking, and it is a commonly staple food. Nowdays, people's requirements for cooked rice focus more on the taste characteristics and quality. Furthermore, aroma is one of the primary sensory reference points, which is the most intuitive way for people to judge cooked rice. By integrating and analyzing the researches of cooked rice aroma identification in recent five years, this paper expounds the extraction and identification methods (sensory evaluation method, GC-MS, SPME, MOS sensors, electronic nose, etc.) of the flavor substances in cooked rice, as the processing methods and properties of cooked rice, and the volatile organic compounds of cooked rice under different conditions are summarized as well.

**Graphical abstract:**

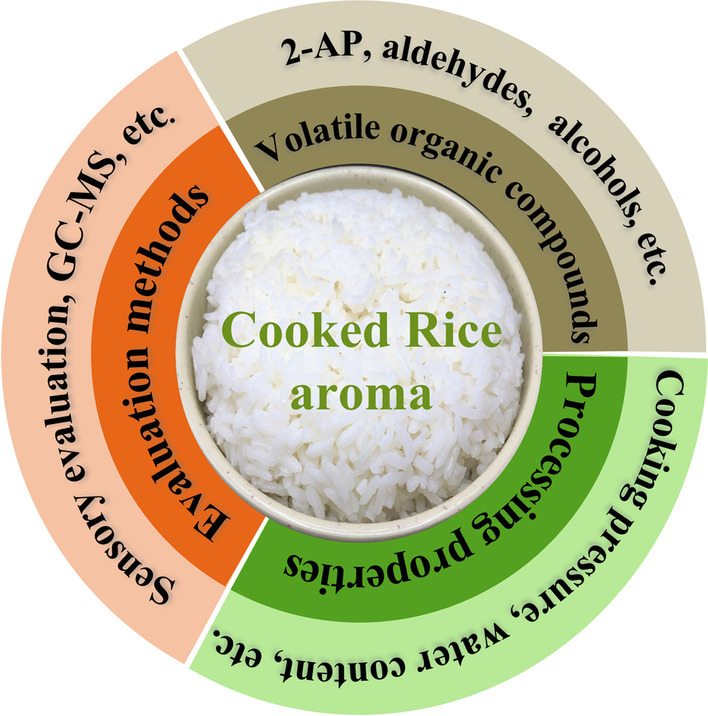

## Background

Rice is a kind of crucial and staple food which feeds more than 4 billion people (Bian et al. 2020; Park et al. 2019). By steaming or boiling, rice can be made into cooked rice which is a daily staple for most Asians (Liu et al. 2021). As a complex carbohydrate rich in protein, sugar, calcium, vitamins, and contains all kinds of amino acids necessary for human beings, the edible and cooking quality of cooked rice has always been the most crucial factor sought by consumers (Timsorn et al. 2017). The degree to which various nutrients are retained in rice grains depends on the post-harvest processing methods, including hulling, milling, and cooking.

The importance of cooked rice to human beings is self-evident. The detection and exploration of cooked rice taste quality are of great significance for promoting the healthy rice market competition and economic development of agriculture, while the aroma of cooked rice largely determines the taste quality of cooked rice. Among the many VOCs, 2-Acetyl-1-pyrroline (2-AP), a primary volatile compound and biomarker in cooked rice, is the focus of researchers. This biomarker was found in both raw and cooked rice, releasing a distinct popcorn flavor (Hinge et al. 2016). In recent years, a large number of researchers have studied the characteristic VOCs and inner formation mechanism of cooked rice aroma, and the specific factors affecting the flavor of cooked rice involved temperature (Ma et al. 2020), steam pressure (Xu et al. 2019), fragmentation degree (Wang et al. 2019b), etc. However, the aroma quality of cooked rice is very complicated due to the complex interaction of a large number of VOCs and the influence of numerous factors in the storage and processing conditions. Before the development of molecular technology, the genetic basis of cooked rice aroma, especially 2-AP, was studied by genetic and molecular mapping techniques. Rice aroma was found to be a highly genetic trait (Wakte et al. 2017) and repeated genetic analyses of different rice cultivars by some researchers have shown that the major aroma traits in rice are controlled by recessive single-gene inheritance, independent of cytoplasmic genes (Berner and Hoff 1986; Bijral and Gupta 1998). Genetic factors are major contributing factors to the formation of cooked rice aroma, and the same cultivar may produce distinct cooked rice flavors on account of diverse planting, processing and storage conditions. This paper concluded the VOCs, evaluation methods and processing properties of cooked rice flavor.

## VOCs for Cooked Rice Flavor

It has been identified that there are more than 300 kinds of VOCs in cooked rice, and the main components are aldehydes, alcohols, ketones, acids, hydrocarbons, esters and heterocyclic compounds (Hashemi et al. 2013; Wakte et al. 2017). These odor components can be formed into three parts from pH: acidic, basic and neutral. From the point of view of volatile compounds, the aroma in cooked rice can be divided into 2-AP, aldehydes, alcohols and phenols and heterocyclic compounds, etc. On the whole, different types of VOCs give cooked rice different flavor characteristics. The alkaline components showed the aroma characteristics of cooked rice and aldehydes imparted a fruity aroma to cooked rice. Alcohols imparted aroma and floral aroma to cooked rice, and esters imparted fruity aroma to cooked rice. For example, nine VOCs including 2-AP, (E, E)-2,4-decadienal, 4-vinyl-guaiacol, nonanal, (E)-2-nonenal, octanal, decanal, hexanal and 4-vinylphenol in cooked rice were identified by Buttery et al and these VOCs are classified as significant contributors to the aroma intensity of cooked rice in the twentieth century (Buttery et al. 1988). In addition, protein, starches and lipids are the three main substances in cooked rice. Although lipids are less abundant in cooked rice than starches and proteins, it positively relates to palatability of cooked rice (Yoon et al. 2012). As a kind of starch, amylose can generate complexes with multiple aroma compound ligands. VOCs produced by main components of cooked rice are presented in Table 1.

**Table 1:** The flavors produced by main cooked rice components

Rice components	Mainly produced substances	Common compounds	Literature
Protein	Aldehydes and ketones, furans, pyrrole and sulfurous compound	2-phenyl ethanol, phenylacetic acid, 6-methyl-5-heptene-2-ketone, 1-pyrroline, 2-methyl-3-furanthiol and dimethyl sulfide	Yoon et al. (2012), Amagliani et al. (2017), Oliveira et al. (2022), Zhang et al. (2021), Monsoor and Proctor (2004), Bryant and McClung (2011), Yang et al. (2008), Grimm et al. (2011) and Jezussek et al. (2002)
Lipids	Aldehydes, ketones, furans, alcohols, acids and hydrocarbons	Hexanal, valeraldehyde, (E)-2-octenal, (E, E)-2, 4-decdienal, 3-pentene-2-ketone, 2-pentylfuran, amyl alcohol, oleic acid, linoleic acid and heptadecane	
Starch	Aldehydes and ketones, furans and pyrrole	Nonanal, butanedione, furfural, 2-acetyl-1-pyrroline	
Maillard-derived volatiles	Pyrazines, Strecker aldehydes, etc	2-methoxy-3,5-dimethylpyrazine, 2-isobutyl-3-methoxypyrazine, benzeneacetaldehyde, 3-methylbutanal, 2-methylbutanal, etc	

Ma et al. studied the relationship between five aromatic compounds (hexaldehyde, 1-octen-3-ol, γ-decalactone, 2-AP, 2,3-butanedione) and amylose in cooked rice. Experimental results showed that except for 2,3-butanedione, the other four aroma components can interact with amylose to form V-type crystal complexes, confirming the effect of amylose on aroma release (Ma et al. 2019). Table 2 summarizes some VOCs released from cooked rice. In general, there are many kinds of VOCs in cooked rice, but only a few of them have an effect on the overall flavor of cooked rice (Champagne 2008). Specifically, 2-AP, aldehydes, heterocyclic compounds and alcohols released from cooked rice were elaborated in details.

**Table 2:** Volatile organic compounds in cooked rice

Volatile organic compounds	Odor description	Extraction method	Cooked rice types	RI				Literature
				DB-WAX	ZB-5	Capillary GC	FFAP	
Hexanal	Leaf-like	SDE, DHS	Cooked korean non-aromatic rice	1078				Park et al. (2010)
	Green	SPME GC-O, GC-PFPD	Jasmine rice (khao dawk mali 105)	1086	799			Mahattanatawee and Rouseff (2014)
	–	GC-O/GC-MS	Japonica cooked rice			1081		Zhao et al. (2022)
	Green	HRGC-O/HRGC-MS	Cooked brown rice				1089	Jezussek et al. (2002)
Unknown	Earthy, sulfury	SDE	Cooked korean non-aromatic rice	1096				(Park et al. 2010)
2-Pentylfuran	Green bean	SDE, DHS	Cooked korean non-aromatic rice	1224				Park et al. (2010)
	–	GC-O/GC-MS	Japonica cooked rice			1227		Zhao et al. (2022)
2-Methyl-3-furanthiol	vitamin, meaty, cooked rice	SDE, DHS	Cooked korean non-aromatic rice	1297				Park et al. (2010)
	Meaty	SPME GC-O, GC-PFPD	Jasmine rice (khao dawk mali 105)	1313	873			Mahattanatawee and Rouseff (2014)
	Meaty, sulfurous	HRGC-O/HRGC-MS	Cooked brown rice				1319	Jezussek et al. (2002)
2-Acetyl-1-pyrroline	Popcorn	SDE, DHS	Cooked korean non-aromatic rice	1340				Park et al. (2010)
	Cooked jasmine rice	SPME GC-O, GC-PFPD	Jasmine rice (khao dawk mali 105)	1342				Park et al. (2010)
	Popcorn, toasted grain, nuty	HS-SPME-GC-MS/MS	Ten cooked rice samples^a^	1354				Kasote et al. (2021)
	-	GC-O/GC-MS	Japonica cooked rice			1353		(Zhao et al. 2022)
	Popcorn-like	HRGC-O/HRGC-MS	Cooked brown rice				1330	Jezussek et al. (2002)
Nonanal	Tallowy	SDE, DHS	Cooked korean non-aromatic rice	1401				Park et al. (2010)
	Green, citrusy, soapy	SPME GC-O, GC-PFPD	Jasmine rice (khao dawk mali 105)	1396	1106			Mahattanatawee and Rouseff (2014)
	Green, fatty, citrus	HS-SPME-GC-MS/MS	Ten cooked rice samples^a^	1401				Kasote et al. (2021)
	–	GC-O/GC-MS	Japonica cooked rice			1396		Zhao et al. (2022)
(E)-2-Octenal	Fatty	SDE	Cooked korean non-aromatic rice	1427				Park et al. (2010)
	Green, nutty	SPME GC-O, GC-PFPD	Jasmine rice (khao dawk mali 105)	1435	1062			Mahattanatawee and Rouseff (2014)
	–	GC-O/GC-MS	Japonica cooked rice			1435		Zhao et al. (2022)
1-Octen-3-ol	Mushroom-like	SDE	Cooked korean non-aromatic rice	1445				Park et al. (2010)
	Green, mushroom, earthy, oily	HS-SPME-GC-MS/MS	Ten cooked rice samples^a^	1455				Kasote et al. (2021)
Methional	Baked potato	SDE, DHS	Cooked korean non-aromatic rice	1451				Park et al. (2010)
	Cooked potato	SPME GC-O, GC-PFPD	Jasmine rice (khao dawk mali 105)	1456	910			Mahattanatawee and Rouseff (2014)
Decanal	Flowery	SDE, DHS	Cooked korean non-aromatic rice	1504				Park et al. (2010)
	Fatty, citrusy	SPME GC-O, GC-PFPD	Jasmine rice (khao dawk mali 105)	1506	1202			Mahattanatawee and Rouseff (2014)
(E)-2-Nonenal	Tallowy, green	SDE, DHS	Cooked korean non-aromatic rice	1545				Park et al. (2010)
	Metallic	SPME GC-O, GC-PFPD	Jasmine rice (khao dawk mali 105)	1540	1161			Mahattanatawee and Rouseff (2014)
Unknown	Earthy	SDE	Cooked korean non-aromatic rice	1626				Park et al. (2010)
(E,Z)-2,4-Decadienal	Waxy, fatty	SDE	Cooked korean non-aromatic rice	1767				Park et al. (2010)
	–	GC-O/GC-MS	Japonica cooked rice			1769		Zhao et al. (2022)
(E,E)-2,4-Decadienal	Waxy, fatty	SDE, DHS	Cooked korean non-aromatic rice	1777				Park et al. (2010)
	Fatty, metallic	SPME GC-O, GC-PFPD	Jasmine rice (khao dawk mali 105)	1820	1318			Mahattanatawee and Rouseff (2014)
	–	GC-O/GC-MS	Japonica cooked rice			1824		Zhao et al. (2022)
Unknown	Vitamin, meaty, cooked rice	SDE, DHS	Cooked korean non-aromatic rice	1897				Park et al. (2010)
4-Vinylguaiacol	Clove	SDE	Cooked korean non-aromatic rice	2196				Park et al. (2010)
Dimethyl sulphide	Cooked, sulfury	SPME GC-O, GC-PFPD	Jasmine rice (khao dawk mali 105)	760	690			Mahattanatawee and Rouseff (2014)
3-Methyl-2-butene-1-thiol	Nutty, sulfury	SPME GC-O, GC-PFPD	Jasmine rice (khao dawk mali 105)	1093	824			Mahattanatawee and Rouseff (2014)
Octanal	Citrusy	SPME GC-O, GC-PFPD	Jasmine rice (khao dawk mali 105)	1290	999			Mahattanatawee and Rouseff (2014)
	Citrus-like	HRGC-O/HRGC-MS	Cooked brown rice				1284	Jezussek et al. (2002)
	–	GC-O/GC-MS	Japonica cooked rice			1295		Zhao et al. (2022)
1-Octen-3-one	Mushroom	SPME GC-O, GC-PFPD	Jasmine rice (khao dawk mali 105)	1303	980			Mahattanatawee and Rouseff (2014)
	Mushroom-like	HRGC-O/HRGC-MS	Cooked brown rice				1300	Jezussek et al. (2002)
Hexanol	Green	SPME GC-O, GC-PFPD	Jasmine rice (khao dawk mali 105)	1376	869			Mahattanatawee and Rouseff (2014)
Dimethyl trisulfide	Sulfury, cabbage-like	SPME GC-O, GC-PFPD	Jasmine rice (khao dawk mali 105)	1384	979			Mahattanatawee and Rouseff (2014)
Unknown	Musty	SPME GC-O, GC-PFPD	Jasmine rice (khao dawk mali 105)	1439	–			Mahattanatawee and Rouseff (2014)
1-Octanol	Fatty, metallic	SPME GC-O, GC-PFPD	Jasmine rice (khao dawk mali 105)	1575	-			Mahattanatawee and Rouseff (2014)
	Waxy, green citrus	HS-SPME-GC-MS/MS	Ten cooked rice samples^a^	1564				Kasote et al. (2021)
	–	GC-O/GC-MS	Japonica cooked rice			1575		Zhao et al. (2022)
(E,Z)-2,6-Nonadienal	Green, metallic	SPME GC-O, GC-PFPD	Jasmine rice (khao dawk mali 105)	1593	1157			Mahattanatawee and Rouseff (2014)
Unknown	Roasted, nutty	SPME GC-O, GC-PFPD	Jasmine rice (khao dawk mali 105)	1635	-			Mahattanatawee and Rouseff (2014)
(E)-2-Decenal	Green herbal geranium	SPME GC-O, GC-PFPD	Jasmine rice (khao dawk mali 105)	1656	1275			Mahattanatawee and Rouseff (2014)
(E,E)-2,4-Nonadienal	Fatty, metallic	SPME GC-O, GC-PFPD	Jasmine rice (khao dawk mali 105)	1711	1218			Mahattanatawee and Rouseff (2014)
Dodecanal	Minty, soapy	SPME GC-O, GC-PFPD	Jasmine rice (khao dawk mali 105)	1727	1419			Mahattanatawee and Rouseff (2014)
2-Acetyl-2-thiazoline	Cooked jasmine rice	SPME GC-O, GC-PFPD	Jasmine rice (khao dawk mali 105)	1766	1112			Mahattanatawee and Rouseff (2014)
Geranyl acetate	Floral	SPME GC-O, GC-PFPD	Jasmine rice (khao dawk mali 105)	1780	1382			Mahattanatawee and Rouseff (2014)
β-Damascone	Sweet honey	SPME GC-O, GC-PFPD	Jasmine rice (khao dawk mali 105)	1828	1425			Mahattanatawee and Rouseff (2014)
β-Damascenone	Sweet honey	SPME GC-O, GC-PFPD	Jasmine rice (khao dawk mali 105)	1833	1395			Mahattanatawee and Rouseff (2014)
α-Ionone	Floral	SPME GC-O, GC-PFPD	Jasmine rice (khao dawk mali 105)	1861	1459			Mahattanatawee and Rouseff (2014)
Unknown	Medicine	SPME GC-O, GC-PFPD	Jasmine rice (khao dawk mali 105)	1867	-			Mahattanatawee and Rouseff (2014)
2-Phenylethanol	Floral	SPME GC-O, GC-PFPD	Jasmine rice (khao dawk mali 105)	1907	1106			Mahattanatawee and Rouseff (2014)
β-Ionone	Raspberry, floral	SPME GC-O, GC-PFPD	Jasmine rice (khao dawk mali 105)	1952	1496			Mahattanatawee and Rouseff (2014)
Ethyl butyrate	Fruity, green, apple, fatty	HS-SPME-GC-MS/MS	Ten cooked rice samples^a^	1046				Kasote et al. (2021)
Ethyl 3-methylbutanoate	Fruity, sweet apple, pineapple	HS-SPME-GC-MS/MS	Ten cooked rice samples^a^	1077				Kasote et al. (2021)
Ethyl hexanoate	Fruity, apple peel	HS-SPME-GC-MS/MS	Ten cooked rice samples^a^	1242				Kasote et al. (2021)
(E)-2-Heptenal	Fruity, green, fatty	HS-SPME-GC-MS/MS	Ten cooked rice samples^a^	1338				Kasote et al. (2021)
1-Hexanol	Green, herbaceous, woody, sweet	HS-SPME-GC-MS/MS	Ten cooked rice samples^a^	1361				Kasote et al. (2021)
	-	GC-O/GC-MS	Japonica cooked rice			1383		Zhao et al. (2022)
Ethyl octanoate	Fruity, fatty, brandy	HS-SPME-GC-MS/MS	Ten cooked rice samples^a^	1444				Kasote et al. (2021)
Ethyl 3-hydroxybutyrate	Green, fruity, waxy, apple skin	HS-SPME-GC-MS/MS	Ten cooked rice samples^a^	1529				Kasote et al. (2021)
2,3-Butanediol	Creamy, fruity, buttery	HS-SPME-GC-MS/MS	Ten cooked rice samples^a^	1548				Kasote et al. (2021)
2-Undecanone	Waxy, fruity creamy, fatty, floral	HS-SPME-GC-MS/MS	Ten cooked rice samples^a^	1606				Kasote et al. (2021)
Ethyl benzoate	Sweet, fruity, wintergreen, medicinal,	HS-SPME-GC-MS/MS	Ten cooked rice samples^a^	1682				Kasote et al. (2021)
Naphthalene	Pungent, tarry	HS-SPME-GC-MS/MS	Ten cooked rice samples^a^	1764				Kasote et al. (2021)
	–	GC-O/GC-MS	Japonica cooked rice			1713		Zhao et al. (2022)
Ethyl benzeneacetate	–	HS-SPME-GC-MS/MS	Ten cooked rice samples^a^	1798				Kasote et al. (2021)
2-Methylnaphthalene	Sweet, floral, woody	HS-SPME-GC-MS/MS	Ten cooked rice samples^a^	1878				Kasote et al. (2021)
1-Methylnaph-thalene	Naphthyl, medicinal	HS-SPME-GC-MS/MS	Ten cooked rice samples^a^	1916				Kasote et al. (2021)
Phenylethyl Alcohol	Floral, sweet, rosey, honey	HS-SPME-GC-MS/MS	Ten cooked rice samples^a^	1929				Kasote et al. (2021)
Ethyl 9-hexadecenoate	–	HS-SPME-GC-MS/MS	Ten cooked rice samples^a^	2267				Kasote et al. (2021)
Indole	Animal, floral, mothball	HS-SPME-GC-MS/MS	Ten cooked rice samples^a^	2475				Kasote et al. (2021)
	–	GC-O/GC-MS	Japonica cooked rice			2441		Zhao et al. (2022)
1-Butanol	–	GC-O/GC-MS	Japonica cooked rice			1135		Zhao et al. (2022)
Pyridine	–	GC-O/GC-MS	Japonica cooked rice			-		Zhao et al. (2022)
Benzaldehyde	–	GC-O/GC-MS	Japonica cooked rice			1531		Zhao et al. (2022)
Acetophenone	–	GC-O/GC-MS	Japonica cooked rice			1615		Zhao et al. (2022)
2-Undecenal	–	GC-O/GC-MS	Japonica cooked rice			1748		Zhao et al. (2022)
Hexanoic acid	–	GC-O/GC-MS	Japonica cooked rice			1855		Zhao et al. (2022)
Benzyl alcohol	–	GC-O/GC-MS	Japonica cooked rice			1855		Zhao et al. (2022)
Heptanoic acid	–	GC-O/GC-MS	Japonica cooked rice			1885		Zhao et al. (2022)
Benzothiazole	–	GC-O/GC-MS	Japonica cooked rice			1938		Zhao et al. (2022)
1-Dodecanol	–	GC-O/GC-MS	Japonica cooked rice			1965		Zhao et al. (2022)
Phenol	–	GC-O/GC-MS	Japonica cooked rice			1998		Zhao et al. (2022)
2-Pentadecanone	–	GC-O/GC-MS	Japonica cooked rice			2016		Zhao et al. (2022)
2-Pyrrolidinone	–	GC-O/GC-MS	Japonica cooked rice			2029		Zhao et al. (2022)
Octanoic acid	–	GC-O/GC-MS	Japonica cooked rice			2088		Zhao et al. (2022)
6,10,14-Trimethyl-2-pentadecanone	–	GC-O/GC-MS	Japonica cooked rice			2116		Zhao et al. (2022)
2-Methoxy-4-vinylphenol	–	GC-O/GC-MS	Japonica cooked rice			2173		Zhao et al. (2022)
Nonanoic acid	–	GC-O/GC-MS	Japonica cooked rice			2183		Zhao et al. (2022)
Methyl palmitate	–	GC-O/GC-MS	Japonica cooked rice			–		Zhao et al. (2022)
Decanoic acid	–	GC-O/GC-MS	Japonica cooked rice			–		Zhao et al. (2022)
2-Tetradecanone	–	GC-O/GC-MS	Japonica cooked rice			–		Zhao et al. (2022)
Ethyl palmitate	–	GC-O/GC-MS	Japonica cooked rice			–		Zhao et al. (2022)
4-Methyl-5-thiazoleethanol	–	GC-O/GC-MS	Japonica cooked rice			2299		Zhao et al. (2022)
2,4-Di-tert-butylphenol	–	GC-O/GC-MS	Japonica cooked rice			2337		Zhao et al. (2022)
2,3-Dihydrobenzofuran	–	GC-O/GC-MS	Japonica cooked rice			2398		Zhao et al. (2022)
Dodecanoic acid	–	GC-O/GC-MS	Japonica cooked rice			2513		Zhao et al. (2022)
Tridecanoic acid	–	GC-O/GC-MS	Japonica cooked rice			–		Zhao et al. (2022)
Vanillin	–	GC-O/GC-MS	Japonica cooked rice			2538		Zhao et al. (2022)
Butan-2,3-dione	Buttery	HRGC-O/HRGC-MS	Cooked brown rice				985	Jezussek et al. (2002)
2-Methoxy-3,5-Dimethylpyrazine	Earthy	HRGC-O/HRGC-MS	Cooked brown rice				1423	Jezussek et al. (2002)
2-Isobutyl-3-methoxypyrazine	Earthy, green bell pepper	HRGC-O/HRGC-MS	Cooked brown rice				1514	Jezussek et al. (2002)
Benzeneacetaldehyde		HSSE/GC/MS	Cooked jasmine rice	1045				Grimm et al. (2011)
3-Methyl-butanal		HSSE/GC/MS	cooked jasmine rice	652				Grimm et al. (2011)
2-Methyl-butanal		HSSE/GC/MS	Cooked jasmine rice	660				Grimm et al. (2011)

*FFAP* free fatty acid phase, *SDE* steam distillation and solvent extraction, *DHS* dynamic headspace sampling, *SPME GC*-*O* solid phase microextraction gas chromatography-olfactometry, *GC*-*PFPD* gas chromatography-pulsed flame photometric detector, *HS-SPME* headspace solid-phase micro-extraction, *HS*-*SPME*-*GC*-*MS/MS* headspace solidphase microextraction with gas chromatography-tandem mass spectrometry, *HRGC/O* high-resolution gas chromatography-olfactometry, *HRGC/MS* high-resolution gas chromatography-mass spectrometry, *HSSE/GC/MS* headspace sorptive extraction gas chromatography-mass spectrometer, *RI* retention index

^a^Ten cooked rice samples: IR-64, Pusa Basmati-1, Pusa Basmati-1509, and Pusa 1652 (Improved Kala Namak), Jeera 32, Govind Bhog, Kala Jeera, Kala Nuniya, Kala Namak-1, and Kala Namak-2;

### 2-Acetyl-1-Pyrroline

2-AP is known as the most representative flavor in determining the overall aroma of cooked rice (Wakte et al. 2017; Wei et al. 2017). It is a significant VOC in raw rice and cooked rice, with a typical popcorn aroma (Mahalapbutr et al. 2021), which is obviously shown in Table 2. The cooked rice aroma can date back to the early 1980s when it was first analyzed and assessed with the description of "popcorn smell". Buttery et al. ranked the popcorn odor intensity of several fragrant cooked rice, among which 2-AP was identified as the most significant contributor to this odor, while it was detected in the range of 6–90 ppb from a series of varieties of cooked rice (Buttery et al. 1983). Tava et al. utilized GC and GC-MS to detect the aromas of Commercial Basmati and Italian Line B5-3, and found that the concentrations of 2-AP were 570 and 2350 ppb respectively (Tava and Bocchi 1999). In general, the concentration of 2-AP in cooked rice was low in ppb level, and it was affected by genetic differences, storage conditions and post-harvest factors (Ma et al. 2020; Li et al. 2022). The concentration of 2-AP is different in various cooked rice. However, 2-AP can be found in all aromatic rices after cooking, but only some types of non-aromatic rice can release 2-AP after cooking (Kasote et al. 2021). With the prolonged of storage time, 2-AP in cooked rice will be oxidized, and the concentration will decrease accordingly (Bryant and McClung 2011). Therefore, Drying and low-temperature storage of harvested rice can effectively increase the content of 2-AP in cooked rice, and improve the taste quality of cooked rice (Liu et al. 2021). Some researchers illustrated that 2-AP was produced by the Maillard reaction after rice is cooked with rising temperature, as the physicochemical reactions between amino acids and carbohydrates or their degradation products in cooked rice will generate 2-AP (Hofmann and Schieberle 1998; Bösl et al. 2021). For instance, the concentrations and odor activity values in cooked basmati rice is 610 μg/kg and 83,516 respectively (Buttery et al. 1994). The 2-AP content of cooked rice is less than that of raw rice, and some 2-AP can be biosynthesised in aromatic and nonaromatic rice crops (Itani et al. 2004; Poonlaphdecha et al. 2016; Prodhan and Shu 2020).

### Aldehydes

The aldehydes (hexanal, nonanal, decanal, octanal, Methional, etc.) are thought to decompose primarily through lipid oxidation which account for the largest proportion of cooked rice flavor content (Table 2). Nonanal, with the RI (Retention Index) of 1401 and concentration of 34.8 ± 0.7a ng/g in cooked rice Pusa Basmati-1509, mainly contributes tallowy green, citrusy, soapy and fatty flavor for cooked rice (Kasote et al. 2021). Meanwhile, hexanal contributes leaf-like and green for cooked rice, and the odour intensity is 9.96 for Basmati cooked rice (Mahattanatawee and Rouseff 2014; Park et al. 2010). After cooking, non-fragrant rice contains significantly more nonanal content than fragrant rice, so cooking can distinguish the two kinds of rice (Park et al. 2010). The odor of cooked rice left over for a long time may be related to the formation of aldehydes. Therefore, Tsuchida et al. constructed a chemical analysis technology for the flavor composition of cooked rice by examining the difference in chemical composition between fresh cooked rice and cooked rice that had been left for a period of time. The bamboo charcoal was used to improve the cooked rice flavor and the chemical evaluation was conducted later. GC-MS analysis showed that the smell of placed rice was connected with the generation of aldehydes such as hexanal, heptanal, nonanal, and octanal in the storage process. These aldehydes were present in the steam before, during, and after cooking the rice. Fourier Transform InfraRed (FT-IR) measurements showed an obvious ester-based peak before cooking, while it did not exist after the rice had been left for some time. This peak appeared due to the formation of a new aldehyde group, indicating partial hydrolysis and oxidation of the esters converted to aldehydes. The findings suggested that the odor of placed cooked rice was closely bound up with aldehydes. Bamboo charcoal could adsorb aldehyde compounds in the cooking duration and reduce the odor of aged cooked rice, which was consistent with the research results of the sensory analysis report (Tsuchida and Kuwahara 2019). A small amount of aldehydes can give cooked rice a pleasant fruity flavor, as the storage time increases, the cooked rice will produce much aldehydes which lead to the formation of odor. In addition, strecker aldehydes (2-methypropanal, 2-methylbutanal, 3-methylbutanal etc.) at parts per million threshold levels produced through maillard reaction contribute malty aroma characteristics primarily for cooked rice (Arsa et al. 2019; Pico et al. 2017; Grimm et al. 2011). Therefore, attention should be paid to the control of the content of aldehydes in cooked rice processing.

### Heterocyclic Compounds and Alcohols

Heterocyclic compounds which consists of monoheterocyclic and gelled heterocyclic compound (furan, Thiazole, pyridine, quinoline, etc.) were identified in Table 2. Specifically, the degradation of starch occurs principally through retroaldol reactions, which generate tiny reactive fragments, such as hydroxylated carbonyl compounds and α-diketo compounds, which act on α-amino acids to organize labile intermediates. Most of the labile intermediates undergo a specific condensation or degradation reaction resulting in heterocyclic substances, some of whom exhibit profound flavor properties. Meanwhile, the heterocyclic flavors of cooked rice found in Maillard flavor compounds include pyridines, thiazoles, pyrazines, furans, oxazoles, and pyrroles and their derivatives. (Demyttenaere et al. 2002). Dihydrobenzofuran, 2-pentylfuran, 2-acetyl-2-thiazoline etc. were produced by lipid reaction and Maillard reaction, and have less overall content in cooked rice (Hoffmann et al. 2019). In furans, 2-alkyl furans with long side chains are usually formed by oxidation of lipids. 2-pentylfuran probably comes from the secondary oxidation products of monohydroperoxides, which typically give cooked rice its nutty and bean flavor (Verma and Srivastav 2020). 2-pentylfuran has been also found in diverse thermally processed foods and juices (Zeng et al. 2009), and it can be detected using deuterated analogs (Frank et al. 2020). The presence of specific amounts of lipid oxidation products, e.g., 2-pentylfuran and indole, can reduce the aromaticity of cooked rice, negatively affecting consumer’s acceptance. Excellent rice varieties usually possess high level of 2-AP and low concentration of lipid-oxidizing compounds.

As shown in Table 2, alcohols (1-octen-3-ol, hexanol, 1-octanol, etc.) obtained a higher odor threshold and they are considered to be the most abundant compound except for aldehydes (Yang et al. 2008). Meanwhile, the floral and fruity aroma of cooked rice are contributed by alcohols (Verma and Srivastav 2020). In addition, among all alcohols, 1-octen-3-ol was thought to be produced in lipid oxidation, it obtains high content and low threshold with the smell of wild mushrooms, contributing the most to cooked rice aroma (Wang et al. 2019b).

## Evaluation Methods for Cooked Rice Flavor

The detection methods of cooked rice flavor can be divided into sensory evaluation method and instrumental analysis method (gas chromatograph (GC), electronic nose (E-nose), metal oxide semiconductor (MOS) sensors, etc.), while the detection results consist of qualitative analysis and quantitative comparison. From the qualitative analysis, the classes of various VOCs can be obtained, and the different rice varieties can be identified or distinguished. The concentration of specific VOCs can be obtained from quantitative analysis.

### Sensory Evaluation Method

Sensory evaluation with human noses as detectors furnishes direct, intuitive, unique and subjective cooked rice flavor information. This method offers the ultimate human sense, while the theoretical odor detection limit of the human nose is around 10^−19^ mol (Wilkie et al. 2004). For the detection of cooked rice flavor, sensory evaluation is usually carried out in eating cooked rice (Lapchareonsuk and Sirisomboon 2014; Limpawattana and Shewfelt 2010). The sensory evaluation method can subjectively evaluate the taste characteristics of cooked rice, and several trained panel members usually carry out the method. They will be requested to assess sensory information about the taste, aroma and overall taste quality of the cooked rice. During the sample assessment, each panelist will consume some specific food (such as natural mineral water and saltless crackers) to maintain a clean taste. The final sensory scores are statistically analyzed to ensure the accuracy of the experiment (Han et al. 2016).

Honma et al. used quantitative descriptive analysis (QDA) for studying the sensory properties of 10 cooked brown rice samples. Eight technical panelists chose 94 sensory-descriptive terminologies to evaluate four samples by open-ended sensory evaluation mode. Preliminary sensory testing identified 18 kinds of evaluation criterion (Aroma: 3; Appearance: 4; Taste: 4; Taste: 1; Texture: 6), as the aroma includes “the smell of green grass after mowing”, “the aroma of freshly cooked white rice” and the aroma of sweet boiled red beans”. After multiple comparisons, 18 attributes showed evident differences (*P* < 0.05) among ten cooked brown rice samples (Honma et al. 2019).

**Fig. 1:**
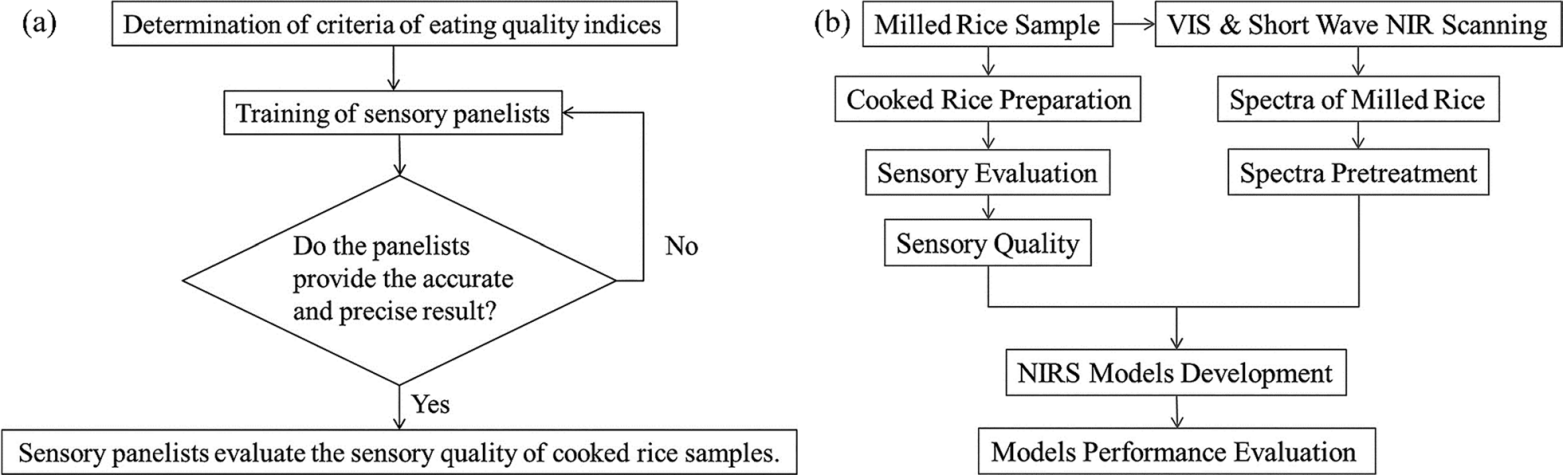
**a** Flow diagram of sensory method for cooked rice; **b** Flow diagram of NIR spectroscopy methodology. Data were obtained from (Lapchareonsuk and Sirisomboon. 2014) (Open Access)

Lapchareonsuk and Sirisomboon et al. proposed to exploit visible light and shortwave near-infrared spectroscopy (NIRS) techniques for analyzing the aroma quality of cooked rice. 4 distinct cultivars of polished rice in this research were utilized: cooked rice, white rice, fresh and aged jasmine rice. The organoleptic quality of the rice, such as stickiness, firmness, dryness, whiteness and aroma, was judged through a sensory team, which turned out that these sensory properties were related to visible light and shortwave NIRS data. The flow chart of sensory evaluation of cooked rice and the flow chart of NIRS are shown in Fig. 1a–b. Data analysis visible and shortwave NIRS models were built using partial least squares regression to predict the sensory quality of cooked rice. The R^2^_val_ values of the sensory quality prediction results all varied from 0.837 to 0.918, among which the cooked rice aroma had the maximum R^2^_val_ value of 0.918 (Lapchareonsuk and Sirisomboon 2014).

### GC Method

GC method is a common method for VOCs identification in cooked rice (Verma and Srivastav 2020; Routray and Rayaguru 2018). It consists two important steps, Sample pretreatment and final identification. Generally, solvent extraction and headspace (HS) method are often used in cooked rice sample pretreatment. The cooked rice aroma are usually subjected to isotopic dilution with the combination of GC-MS and the detection standards of each VOC is individually set to quantify the detected substances accurately (Frank et al. 2020).

Shi et al. employed a combined sensory evaluation technique, incorporating gas chromatography-tandem mass spectrometry (GC-MS/MS) and scanning electron microscopy (SEM) to study the variation in aromatic constituents and microstructure of cooked brown rice in the roasting process. Roasting, a powerful processing technique for increasing the content of aromatic compounds in grains, has been extensively applied in rice and cereal products in recent years (Youn and Chung 2012). GC-MS analysis showed that a total of 11 VOCs were determined in cooked brown rice samples, and the roasting raised the content of heterocyclic compounds and resulted in a decrease in the types and contents of hydrocarbons and benzene derivatives (Shi et al. 2018). The primary flavor substances in roasted brown rice are mainly Maillard-derived volatiles included furfural, 5-methylfurfural, 2,5-dimethylpyrazine, 2-methylpyrazine, 2-ethyl-6-methylpyrazine and 2-ethyl-3,5-Dimethylpyrazine. In addition, roasting can lead to an uneven surface of cooked brown rice, increase the space between particles, and promote the production and distribution of aromatic components. Such microstructural variation increases the spillover pathways of aromatic compounds. Qi et al. studied the impacts of various processing methods on the fluidity, solubility, nutritional components, composition, color and aroma components of four types of ready-to-eat brown rice. GC-MS methods obtained quantitative analysis of 18 VOCs, incorporating 13 aldehydes, 2 heterocyclic furans, 1 each of ketones, olefins and acids, and no alcohols and esters were detected (Qi et al. 2019).

**Fig. 2:**
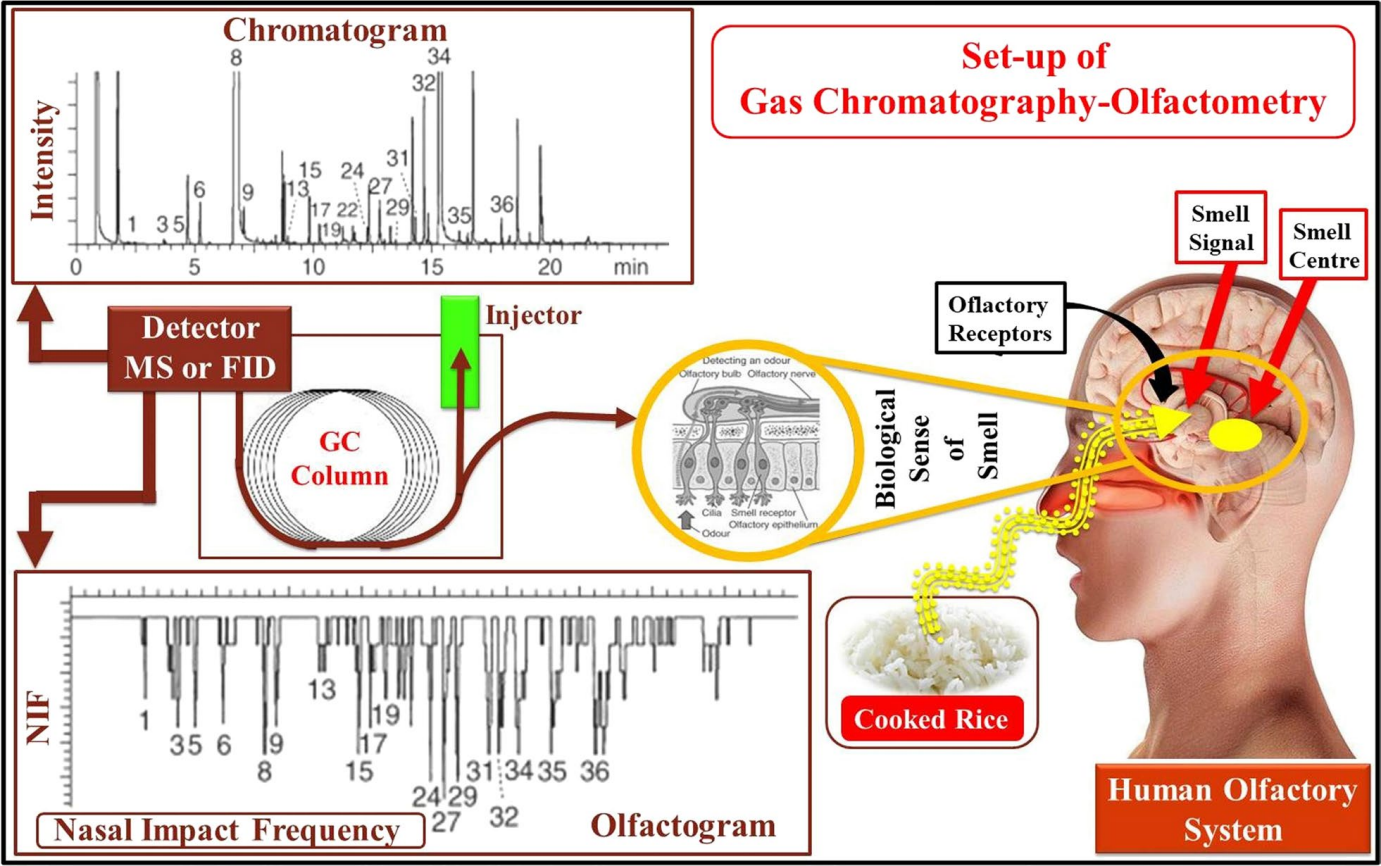
Schematic diagram of GC-O system. Data were obtained from (Verma and Srivastav 2020). Copyright 2019 Elsevier

Dias et al. studied the VOCs produced from fragrant cooked rice (IAC 500) to identify compounds that act a pivotal part in cooked rice flavor. The aroma of IAC 500 rice was mainly described via a carefully selected professional sensory panel, and the VOCs of cooked rice extracted by SPME were identified by olfactometry (OSME) and GC-MS. 80 volatiles in total were identified through OSME, and 65 compounds in total were detected via the chromatographic analysis, while 44 with certain odor and 36 without odor. The detected compounds were mainly aldehydes, alkanes, carboxylic acids, alcohols and ketones (Dias et al. 2021). The study by Maraval et al. aimed to complete the analysis of flavor VOCs of cooked rice released by two fragrant rice samples (Aychade and Fidji) from the Camargue region and compare them with those of a well-known Asian fragrant rice cultivar (Thai). In addition, non-aromatic varieties from the Camargue region were compared with aromatic varieties. The gas chromatography-olfactometry (GC-O) technique was employed to research the odor profile (Utz et al. 2022) of cooked rice samples as it allows the selection and analysis of odorant-active compounds in complex mixtures (López-Galilea et al. 2006; Pozo-Bayón et al. 2007), and the working principle is shown in Fig. 2. In addition, It was more accurate to combined with GC-MS to detect various cooked rice flavor compounds (Maraval et al. 2008).

Furthermore, Gas Chromatography-Olfactometry-Mass Spectrometry (GC-O-MS) integrates the virtue of sensory description analysis and GC-MS. GC-O-MS plays a crucial role in finding pivotal, aromatic and vigorous compounds of cooked rice, providing not only minute sensory evaluation of flavor quality, but also volatilization quantitative and qualitative tests of compounds. Steam distillation and solvent extraction (SDE) is widely used in cooked rice flavor identification. However, it is time-consuming and tedious, and its reproducibility is poor, so it is easy to lead to errors in the analysis process and loss of some compounds (Park et al. 2010).

Zeng et al. used an improved HS-SPME method to directly extract the aroma volatile compounds from 3 Japanese rice varieties, namely Nihonbare, Koshihikari and Akitakomachi in the cooking process, and the GC-MS was used to analyze it. 46 VOCs in total were detected, incorporating aldehydes, ketones, alcohols, heterocyclic compounds, fatty acid esters, phenolic compounds and hydrocarbons. With the prolonging of cooking time, the number of significant odor compounds added while the quantity of volatiles with low boiling points lessened. Guo et al. used headspace-gas chromatography-time-of-flight mass spectrometry (HS-GC-TOF MS) combined with headspace-solid phase microextraction-gas chromatography-time-of-flight mass spectrometry (HS-SPME-GC-TOF MS) for precise and rapid quantification of 2-AP in cooked rice (Guo et al. 2020).

Aseptic-packaged cooked rice (APCR) is a kind of rice food with a promptly expanding market scale. Compared with rice steamed in electric rice cookers, APCR has a longer shelf life (6 months) and only needs to be microwaved for 2 min before eating (Kwak et al. 2015). Lee et al. used a standard addition method combined with SPME/GC-MS to analyze 2-AP flavor in APCR quantitatively. The results manifested that the content of 2-AP in APCR containing 20% fragrant rice stored at 25 ℃ for one month and two months were 15.3– 9.5 ng/g and 6.1 ng/g respectively, which proved the practicability of the method (Lee et al. 2019b).

### MOS Sensors

MOS gas sensor is mainly used to detect the resistance changes of the material after the device contacts with the target gas (Liu et al. 2022). It can quickly detect the single VOC in the cooked rice (Table 3). So far, many kinds of MOS materials, including NiMoO_4_ (Yin et al. 2020), CuCrO_2_ (Zhao et al. 2020), La_2_O_2_CO_3_ (Ding et al. 2017), Bi_2_WO_6_ (Cao et al. 2020) and Sb_2_WO_6_ (Yang et al. 2016) have been broadly utilized in the preparation of gas sensors on account of their easy preparation, reduced energy consumption, low cost, fast response and excellent stability.

**Table 3:** Detection of characteristic flavor in rice based on MOS gas sensor

The target gas	Gas sensor material	Gas concentration (temperature or relative humidity)	Response to the target gas (sensitivity)	Literature
Nonanal	Pt, Pd, Au/SnO_2_	9.5 ppm	59	Itoh et al. (2016)
	SnO_2_ nanosheet	300 ppb (250℃)	2	Masuda et al. (2019)
	Single-crystalline ZnO nanowire	2.48 ppm nonanal in N_2_ (200℃)	6.2	Wang et al. (2020)
	Ru-loaded urchin-like W_18_O_49_ hierarchical nanostructure	10 ppm (RT)	4.79	Zhang et al. (2022)
	Hierarchical Sb_2_WO_6_ microspheres	30 ppm (RT)	62.0	Zheng et al. (2022)
Hexanal	Ti_3_C_2_T_x_-TiO_2_	10 ppm (RT)	3.4	Kuang et al. (2021)
	SnO_2_ nanostruture	100 ppb (350℃)	2.8	Huang et al. (2013)
Propanal	Cubic In_2_O_3_	50 ppm (25℃/50% RH)	71	Sahm et al. (2007)
	ZnO tetrapods	5 ppm (400℃/30% RH)	21	Calestani et al. (2011)
	ZnO tetrapods	50 ppm (400 ℃/30% RH)	73	Calestani et al. (2011)
Ethanol	Zn_2_SnO_4_ nanoparticles/reduced graphene oxide (ZTO/RGO) nanocomposites	100 ppb (250℃)	38	Li et al. (2018)
	Zn_0.9_Mg_0.1_SnO_3_	500 ppm (220℃)	273.46	Wang et al. (2019a)
1-octen-3-ol	SnO_2_	50 ppm (250℃)	10.9	Shokrzadeh et al. (2020)
	SnO_2_/Pd	50 ppm (250℃)	15.1	Shokrzadeh et al. (2020)

#### Aldehyde Detection

Nonanal, hexanal and propionaldehyde are the aldehydes released in large amounts in cooked rice. As a large molecular weight VOC, nonanal (C_9_H_18_O) faces the problem of long recovery time in detection. Itoh et al. used a SnO_2_ gas sensor loaded with Pt, Pd, and Au to detect nonanal. Using the aged Pt, Pd, Au/SnO_2_ gas sensor to detect 9.5 ppm nonanal at 250°C, a response value of 59 can be obtained, but the recovery time was longer (> 300 s) (Itoh et al. 2016). Masuda et al. used a SnO_2_ nanosheet-based gas sensor to detect nonanal vapor at ppb level. Its response to nonanal was higher than carbon monoxide (CO), nitrogen dioxide (NO_2_), acetone (CH_3_COCH_3_), hydrogen (H_2_), ethanol (C_2_H_6_O), ammonia (NH_3_), hydrogen sulfide (H_2_S), formaldehyde (HCHO), acetaldehyde (CH_3_CHO) and butyraldehyde (C_4_H_8_O). At the same time, when the nonanal concentration was increased from 100 to 300 ppb, the response value increased from 1.383 to 2.00 (Masuda et al. 2019). Zhang et al. prepared a Ru-loaded sea urchin-type W_18_O_49_ gas-sensing material, and performed room temperature sensing tests on nonanal which is a chain aldehyde compound produced during rice aging. The gas sensor response of W_18_O_49_ loaded with 1.0% Ru to 30 ppm nonanal reached 16.1 under room temperature, 6.8 times higher than that of pure W_18_O_49_ gas sensor. The improved sensing performance was primarily caused by the sea urchin-like morphology, abundant oxygen defects and the synergistic effect between Ru inhibition (Zhang et al. 2022).

In cooked rice, hexanal is the product of oxidative degradation of oil, and it can emit a pleasant fruity aroma when the concentration is low. Gas sensors based on metal oxides (e.g., ZnO, SnO_2_, and In_2_O_3_) have been widely used to detect hexanal in the past few decades (Huang et al. 2013). The concentration level of hexanal was low in biological samples, ranging from picomolar to micromolar (Li et al. 2005; Chen et al. 2019). The progression of gas sensors that can detect hexanal at low concentrations has become an urgent problem to be solved. Delin et al. used a VOCs gas sensor based on Ti_3_C_2_T_x_-TiO_2_ nanocomposite to detect hexanal at room temperature, and it had a 3.4% response to hexanal at a concentration of 10 ppm. The sensor retained a high signal-to-noise ratio during detection, which possessed the lowest detection limit of 217 ppb for hexanal gas. Huang et al. prepared SnO_2_ nanomaterials based on a hydrothermal method, and assembled a planar coplanar nanoscale SnO_2_ hexanal gas sensor array by screen printing technology. The obtained gas sensor array had good gas-sensing performance with low detection limit and high sensitivity. The response to 100 ppb hexanal gas reached 2.8 at 350℃ (Huang et al. 2013).

#### Alcohols Detection

Alcohols are the second most abundant in cooked rice. Li et al. prepared Zn_2_SnO_4_ nanoparticles with different contents of reduced graphene oxide (RGO) by solvothermal method combined with an annealing process. The gas sensor assembled with materials with a mass ratio of 8:1 (Zn_2_SnO_4_: RGO = 8:1) was prepared. The sensor's response to 100 ppm ethanol at an optimum working temperature of 275°C was up to 38 (Li et al. 2018). Lee et al. prepared pure In_2_O_3_ and In_2_O_3_ nanofiber materials doped with 0.05, 0.1, 0.3, and 0.5 at% Fe. Sensor arrays assembled from these five materials were used for para-benzene, xylene, toluene, formaldehyde and ethanol detection. Gas sensors doped with 0.05 and 0.1 at% Fe showed more significant responses to aromatic VOCs (benzene, xylene, and toluene) and smaller responses to non-aromatic VOCs (ethanol and formaldehyde) (Lee et al. 2019a). Shokrzadeh et al. synthesized SnO_2_ and SnO_2_/Pd nanoparticles (NPs) MOS materials by reducing Pd^2+^ under glycine. Through static process test of 1-octen-3-ol, the results showed that SnO_2_/Pd gas sensor exhibited higher sensitivity than SnO_2_. The responses of SnO_2_/Pd and pure SnO_2_ NPs sensors to 700 ppm target gas were 46.7% and 24.2% under 250°C respectively. The linear segments of the detection limit and quantification limit of the SnO_2_/Pd gas sensor for measured gas at 250℃ reached 20.94 ppm and 69.79 ppm respectively (Shokrzadeh et al. 2020).

#### Other Types of Detection

2-pentylfuran is a VOC with a molecular weight of 138 g/mol and a vapor pressure of about 160 Pa at 25°C (Bhandari et al. 2011). 2-pentylfuran is a furan substance abundant in cooked rice and 2-AP is one of the most crucial VOCs in cooked rice flavor, but no researchers have used MOS gas sensors to measure them (Russo et al. 2014). However, the MOS gas sensor can be combined with machine learning methods to assemble a system to detect 2-pentylfuran and 2-AP in cooked rice, which is more sensitive to changes in gas content.

### Electronic Nose

Electronic nose is a novel instrument for fast food detection developed in the 1990s. It uses diverse gas sensors and pattern recognition systems to provide overall information quickly about the target sample are the corresponding data are distinguished accurately (Zheng and Zhang 2022). MOS gas sensor based E-noses have chemical imaging capabilities and sensor systems with the advantages of excellent cross-sensitivity and fast, stable and broad-spectrum response, which can be used to analyze VOCs in cooked rice (Feng et al. 2011; Jiang et al. 2017).

Jana et al. introduced an aroma-based detection and classification instrument for aromatic rice varieties. It mainly consisted of an odor processing module, an olfactory detection module, a water bath module and a computing module. The odor processing module mainly transfers the flavor of cooked rice to the olfactory detection module. The olfactory detection module is formed by a printed circuit board assembled with eight gas sensors and a sensor chamber. The water bath module, which is connected to a heater to facilitate cooking, is used for preparing rice samples. The computational module quantifies the smell massages collected via the sensors. Principal Component Analysis (PCA) enables data acquisition from sensor arrays and clustering. Probabilistic Neural Network (PNN), Back propagation multilayer perceptron (BPMLP) and Linear Discriminant Analysis (LDA) were used to identify and classify rice varieties (Jana et al. 2015).

Sinelli et al. used E-nose and Fourier transformation near infra-red (FT-NIR) technology for exploring the optimal cooking time (OCT) of unpolished rice, cooked rice and instant rice and compared it with the gelatinization time and suggested cooking time (SCT) on the packaging label. It was found that FT-NIR method could accurately confirm the OCT of polished and cooked rice. Besides, the maximum rate of aromatic flavor change in cooking as assessed via E-nose was associated with the SCT of the 3 rice samples. In addition, the combination of the two methods is more fast, simple, and objective, making it ideal for sensory analysis and rice gelatinization time determination (Sinelli et al. 2006). Rok et al. used an E-nose assembled with 12 gas sensors to study complex odor emitted by 44 japonica rice varieties and characterized the VOCs emitted from japonica rice and cooked japonica rice. The response of gas sensors was evaluated by PCA and clustering analysis (CA) (Song et al. 2005).

In addition to traditional gas sensors, E-noses are currently combined with mass spectrometry and colorimetric sensors, which increases their innovation and reliability in application for cooked rice aroma detection. From what has been discussed above, this paper summarizes the the evaluation techniques for cooked rice flavor, as shown in Table 4 and 5.

**Table 4:** Summary of evaluation methods for identifying VOCs in cooked rice

Evaluation method	Food type (cooked rice)	The main vocs	Cooking method and instrument	Literature
HS-SPME/GC-MS	Brown rice	Hexanal, nonanal and 2-pentylfuran	The high pressure cooking (60, 70 and 105 kpa), the low pressure cooking (30, 40 and 50 kpa)	Yu et al. (2021)
GC	Soybean-rice mixture	–	Cooked by two types of rice cookers: an erc (lj-mg0402) and an eprc (ljp-sa063e)	Kim et al. (2015)
HS-SPME/GC-MS	Three japanese rice cultivars, nihonbare, koshihikari, and akitakomachi	Indole and 2-acetyl-1-pyrroline	Cooked by sra18h automatic electric rice cooker during four stages	Zeng et al. (2008)
SPME/GC-MS	The akitakomachi cultivar of paddy rice (*Oryza sativa* L.)	n-nonanal and hexadecanoic acid	Cooked by sra18h automatic electric rice cooker during four stages	Zeng et al. (2007)
GC-MS/FT-IR	Koshihikari (polished rice)	Hexanal, heptanal, octanal, nonanal	Cooked by ih rice cooker (sr-spx104, panasonic)	Tsuchida and Kuwahara (2019)
Sensory evaluation/E-nose/ HS-SPME/(GC-MS/MS)/SEM	Brown rice (japonica rice)	Furans and pyrazines	roasted by different time and temperature	Shi et al. (2018)
E-nose	Kaminibhog, radhunipagal, govindobhog, sitabhog et al	–	Cooked at 100 °C for 20 min	Jana et al. (2015)
HS-GC-TOF MS/ HS-SPME-GC-TOF MS/ Sensory evaluation	Eight types of rice samples (zhongzao 39, yuexiuyou 376, nanjingxiangzhan et al.)	2-acetyl-1-pyrroline	Cooked according to the agricultural industry standard of china ny/t 596–2002 “aromatic rice” with some modification	Guo et al. (2020)
OSME/GC-O/ SPME/GC-MS	Aromatic rice iac 500	2-acetyl-1-pyrroline	Cooked in an electric pan (mondial, são paulo, brazil) with water at a ratio of 1:2.5 (100 g rice/250 ml water)	Dias et al. (2021)
FGC E-Nose/HS-SPME-GC-MS	Rice produced by yihai kerry co., ltd. (wuchang, heilongjiang, china)	2-acetyl-1-pyrroline and aldehydes	Cooked using an automatic rice cooker (cfxb50fc8055-75, zhejiang supor limited by share ltd., hangzhou, zhejiang, china)	Ma et al. (2020)
GC-MS/GC-O	Three scented cultivars (aychade, fidji, and giano) and a common nonscented cultivar (ruille)	2-acetyl-1-pyrroline, hexan-1-ol, indole and phenol	Rice (5 g) and mineral water (volvic, 10 ml) were cooked in open steam for 20 min	Maraval et al. (2008)
Sensory evaluation /QDA	Brown rice	–	Cooked using an ih rice cooker (sr-fd106, panasonic corporation) without soaking	Honma et al. (2019) and Sinelli et al. (2006)
FT-NIR spectroscopy/E-nose	Milled, parboiled and quick-cooking rice (*Oryza sativa* L. japonica)	–	Soaked in 1650 ml of water and cooked in a rice cooker for times up to 1320, 1200 and 900 s, respectively	Sinelli et al. (2006)
HS-SPME/GC-MS	Aseptic-packaged cooked rice	2-acetyl-1-pyrroline	Microwaved (model kr-g20ew; daewoo electronics co., south korea) for 120 s at 700 w	Lee et al. (2019b)
Sensory evaluation/Shortwave NIR	Jasmine rice	–	Home electronic rice cookers (rc-10 mm; toshiba, thailand)	Lapchareonsuk and Sirisomboon. (2014)
SDE/GC/GC-MS	Rice (*Oryza sativa* L. japonica) harvested in niigata prefecture, japan	–	Cooked in an aluminium cup by an automatic electric rice cooker (toshiba model rc-4b)	Tsugita et al. (1983)
GC-O/GC-MS	Twenty-six japonica rice varieties	2-acetyl-1-pyrroline	Cooked according to a national standard method gb/t 15,682–2008	Zhao et al. (2022)
SDE/DHS/GC-MS	Korean non-aromatic rice	2-methyl-3-furanthiol and 2-acetyl-1-pyrroline	Boiling by adding 200 ml deodorized distilled water	Park et al. (2010)
HS-SPME-GC-MS/MS	Ten cooked rice samples	2-acetyl-1-pyrroline, ethyl butyrate, ethyl 3-methylbutanoate, ethyl benzoate and 2-methylnaphthalene	Boiling by adding 0.25 ml of ultra-pure water with 1 g rice grain sample	Kasote et al. (2021)
SPME GC-O, GC-PFPD	Jasmine rice (khao dawk mali 105)	2-acetyl-1-pyrroline	Cooked in a rice cooker (black & decker, model no. rc3406) at 100 °C, for 18 min	Mahattanatawee and Rouseff (2014)

*HS-SPME* headspace solid-phase micro-extraction, *GC* gas chromatography, *GC*-*MS/MS* gas chromatography-tandem mass spectrometer, *SEM* scanning electron microscope, *HS-GC-TOF MS* headspace-gas chromatography-time-of-flight mass spectrometry, *HS-SPME-GC-TOF MS* headspace-solid phase micro-extraction-gas chromatography-time-of-flight mass spectrometry, *OSME* olfactometry, *GC-O* GC olfactometry technique, *GC-FID* gas chromatograph-flame ionization detector, *FGC E-Nose* flash gas chromatography electronic nose, *HS*-*SPME*-*GC*-*MS* headspace solid-phase micro-extraction method combined with gas chromatography-mass spectrometry, *QDA* quantitative descriptive analysis, *FT-NIR* fourier transformation near infra-red, *NIR* near-infrared spectroscopy

**Table 5:** Overview of detection methods of characteristic flavor in cooked rice

Detection method	Advantage	Disadvantage	Reference for cooked rice (Author and year)
GC-MS	High sensitivity, strong qualitative ability and convenient daily maintenance	Relative high cost for purchase and restriction for widely use as the operation temperature limit (Gruber et al. 2020; Valdez. 2021)	Zeng et al. (2008), Zeng et al. (2009), Tsuchida and Kuwahara. (2019), Yu et al. (2021)
HS-SPME	Low detection limit, relative high sensitivity, simplicity, speed, wider compound coverage, and higher throughput, simple operation and fast test speed, basically no solvent, little environmental pollution, less sample consumption	Sulfur and sulfide compounds cannot be detected (De Giovanni and Marchetti. 2020)	Zeng et al. (2008) and Zeng et al. (2009)
E-nose	Low cost, accurate, fast, reliable and portble	Data collection are tedious and labor intensive, data collection from different sources, the e-nose performance is highly affected by temperature modulation (Al-Dayyeni et al. 2021; Lei and Zhang. 2015)	Chen et al. (2021) and Jana et al. (2015)
GC-O-MS	Identification of key aroma-active compounds accurately, capable of illustrate relationship between odorants and sensory properties	Limited scope of application, need to train professionals to operate (Song and Liu. 2018)	(Hu et al. 2020)
Sensory evaluation	With subjective characteristics	Low reproducibility and poor accuracy	Guo et al. (2020) and Ma et al. (2020)

*GC-O-MS* gas chromatography-olfactometry-mass spectrometry

## Processing Properties for Cooked Rice Flavor

**Fig. 3:**
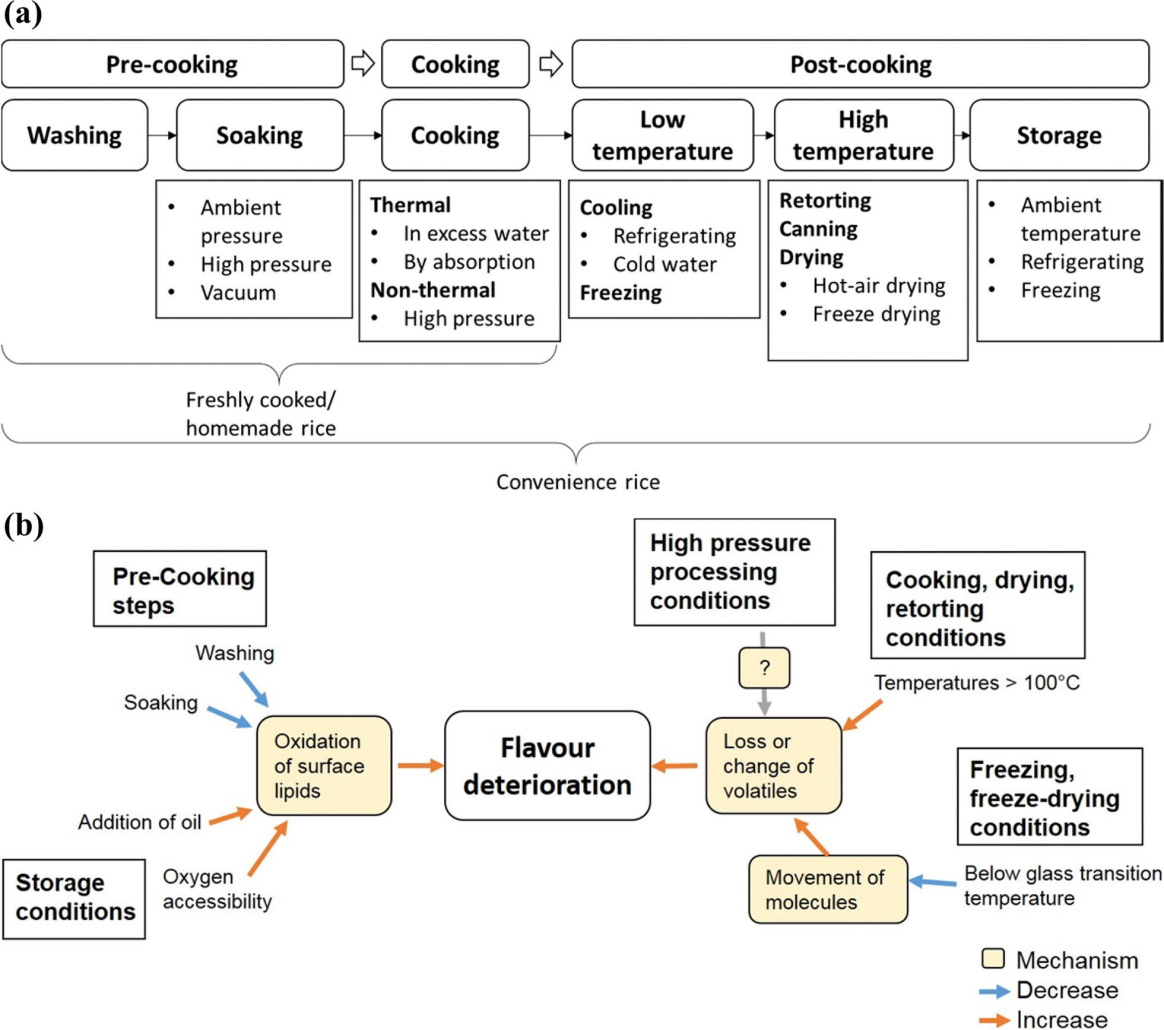
**a** Pre-cooking, cooking and post-cooking technologies with different classifications to produce freshly cooked and convenience rice; **b** Effects of processing conditions on cooked rice flavor deterioration, measured by sensory analysis and/or instruments. Data were obtained from (Yu et al. 2017). Copyright 2017 Elsevier

The whole cooking process is usually broken down into 4 phases: (I) 25 min from the beginning of heating until the steam comes out; (II) the steam starts to come out of the pot and ends 13 min later; (III) the remaining steam overflows from the rice cooker until it stops automatically 10 min of heating; (IV) 30 min of heat preservation starting from automatic stop heating (Zeng et al. 2008). In addition, rice cooking is a gelatinization process: the starch absorbs water and expands when heated, and then the starch is released from the cell wall, destroying the previous crystal structure and forming a gel. The difference in the starch of cooked rice can affect the volatilization of aroma compounds (Bagchi et al. 2021). Proper cooking process can minimize the nutrient loss of food and improve the edible characteristics and quality (Yin et al. 2014), and if the cooking methods are unbefitting, the excessive nutrient may lose and edible quality of cooked rice could decrease. Cooking rice usually requires five steps, namely washing rice, adding water, flooding, heating and heat preservation. The purpose of each step of cooking rice is shown in Table 6 and the last three steps need to be paid more attention for the flavor of cooked rice. Different processing properties of cooked rice greatly impact the VOCs produced (Fig. 3).

**Table 6:** The purpose of each step of cooking

Steps	Purpose	Literature
Wash rise	Remove impurities and odors from rice grains, reduce potential arsenic or toxic metal contaminants	Liu et al. (2018) and Menon et al. (2021)
Add water	Provides water for starch gelatinization	
Flooding	Make rice grain water absorption uniform, conducive to heating gelatinization	
Heating	Provides the energy needed for starch gelatinization	
Heat preservation	Use the remaining temperature in the pot to balance the water between rice grains, and make the starch gelatinize evenly

The aroma of cooked rice is affected by the type of rice, pre-harvest conditions, post-harvest conditions and processing properties (Champagne 2008), as we focus on the effects of some vital processing properties (cooking pressure, water content, temperature, etc. (Bello et al. 2015)) on cooked rice aroma in this section.

### Pressure

Among most Chinese, ordinary and pressure cooking are the universal home processing methods, while pressure cooking can bring better taste (Kim et al. 2015). Xu et al. analysed cooked rice aroma using high pressure steam (HPS) under different levels. The results showed that HPS cooking had significant effects on the flavor characteristics of cooked rice. When the pressure of cooking gradually increased from 0 Mpa to 0.18 Mpa, the content of aldehydes increased, and the content of alcohols and some heterocyclic compounds increased first and then decreased. For example, the content of nonanal raised from 5.789% (0 Mpa) to 7.009% (0.18 Mpa) and 2-pentylfuran’s level went from 3.819 to 10.106%, then down to 8.800% (Xu et al. 2019).

### Water Content

Depending on the different cultural backgrounds, rice varieties and cooking methods, there are two main ways to make cooked rice at home (Yu et al. 2017): (1) steaming with adsorbed pre-determined doses of water; (2) cooking with extra water at the specific temperature (higher than gelatinization temperature). The former method is more prone to insufficient water diffusion through the rice grain. The starch in the center of the granule may not be completely gelatinized during cooking, which will generate a harder texture (Seki and Kainuma 1982). Larger food industry usually uses excess water to cook rice, because a continuous cooking process is obtained, in which the rice particles can be full of moisture with a uniform distribution. Additionally, there are two mechanisms in rice cooking: (1) the water is absorbed asymptotically from the surface to the inside of the rice; (2) the textural composition of grains is changed by heating with water (Xie et al. 2019). As for rice aroma, 11 rice varieties were soaked in water for 30 min, and a negative effect on flavor and sweetness indicates that soaking water could lose a small amount of flavor-active metabolites, while the flavor changing was not related with the change of grain structure (Champagne. 2008; Calingacion et al. 2012). By adjusting the cooking mechanism of rice, the cooking method can be improved and the flavor quality of rice can be further promoted.

The moisture also affects the flavor quality of instant cooked rice. Dehydrated rice and non-dehydrated rice are the two main categories of instant cooked rice. Among them, dehydrated rice (α-rice) is widely popularized due to its convenient use, low moisture content, long shelf life, low and straightforward production process. However, the processed instant cooked rice loses too much flavor and reduces the taste quality. The high quality of the finished instant cooked rice can be determined by its taste character and it needs to be enhanced (Wahengbam et al. 2019, 2020). It is crucial to explore the impacts of different cooking methods on the change of the flavor substance and quality of instant cooked rice after rehydration.

### Temperature

Temperature can indirectly affect the flavor of cooked rice by changing the degree of lipid oxidation. Through reducing the temperature, rapid cooling helps reduce starch degradation during the storage of cooked rice (Yu et al. 2010), which can add much freshness to the cooked rice.

Ma et al. investigated the relationship between cooling rates (CR) (0.19, 1.27, 1.74 and 2.88°C/min) and the cooked rice flavor during the period of storage. The experimental results of flash gas chromatography electronic nose (FGC E-nose) and sensory analysis indicated that the faster the cooling rate was, the longer the aroma retention time of the rice was, while the lower the cooling ratewas, the faster the acceptability of the cooked rice flavor degraded. In the experiment of 17 cooked rice samples in total, the control group (freshly boiled rice) had the top total score, drastically higher than the other samples (*P* < 0.05). The scores for each sample had significant discrepancies (*P* < 0.05) at different storage times. With prolonged storage, the flavor of the samples deteriorated and consumer acceptability decreased (Ma et al. 2020).

The half-boiling method is a hydrothermal method widely used in rice cooking. It causes variation in the physicochemical and nutritional features of rice particles, which has a huge impact on sensory and other taste qualities. The binding between proteins and flavor substances in cooked rice is mainly through electrostatic interactions such as van der Waals forces, hydrogen bonds and disulfide bonds. High temperature may weaken or disappear these forces, leading to the change of flavor substances (Rocha‐Villarreal et al. 2018). Therefore, hydrothermal processing methods during half-boiling can not only passivate lipases in cooked rice, but also inhibit off-flavor production, which significantly enhances the taste quality of cooked rice.

## Conclusions

Rice will release flavor compounds generated via thermal decomposition and Maillard reaction during the cooking process, which has a strong flavor. Over 300 volatile and semi-volatile compounds have been found in cooked rice. However, no significant relationship between these compounds and aroma has been found. 2-AP, aldehyde, heterocyclic and alcohol compounds have a vital role in the fragrance quality of cooked rice. The application of sensory analysis, gas chromatography, and E-nose method in cooked rice aroma analysis was expounded, and the impacts of processing properties and storage conditions on cooked rice samples are indicated. GC is employed for qualitative and quantitative analysis of VOCs, and solvent extraction headspace method is usually adopted for sample pretreating. GC-O-MS can not only achieve a minute sensory evaluation of cooked rice aroma, but also perform accurate quantitative detection of VOCs. E-nose is commonly utilized to categorize various rice samples. MOS gas sensors can detect a single gas in cooked rice, and explore the changes of volatile gas concentration during the cooking process with different cooking methods. Washing, high static pressure, roasting and half-cooking are effective processes to ameliorate the cooked rice aroma. The degree of milling and storage conditions also have grave impacts on the flavor of cooked rice (Hu et al. 2020).

There are still some bottlenecks/challenges in the detection of cooked rice flavor:

1. At present, qualitative measurements such as E-nose have been extensively used, and it can utilize flavor characteristics of the cooked rice o classify rice types. Due to the complexity of cooked rice flavor and the limitations of current sensor technology, there are few fast and non-destructive cooked rice flavor detection instruments that can be used for commercial application. The accurate quantitative characterization technology for flavor dectection is also in urgent need.
2. Different proportions of volatiles in cooked rice may lead to different perception results, mainly the intensity and attributes of perceived odor. The relationship between VOCs and perceived odor has not been well established currently. The relationship between typical VOCs and sensory evaluation results needs to be clarified.
3. There are many flavor substances in rice-derived foods, such as rice cakes, rice wine, rice noodles, rice juice and various foods with rice flavor, which usually need to be fermented or enzymatically hydrolyzed to produce more varieties and contents of aroma substances. Odor detection methods have been put forward with higher requirements.
4. In terms of the qualitative and quantitative detection of substance, different detection methods sometimes produce a significant difference in the detection of cooked rice flavor. The difference is likely to be connected with the significant differences in the pretreatment conditions of rice samples. It is necessary to establish a set of objective, unified and accurate cooked rice flavor detection technology to improve the cooking method of rice and ameliorate the taste quality of cooked rice.

## Data Availability

Not applicable.

## Abbreviations

2-AP: 2-Acetyl-1-pyrroline
FFAP: Free fatty acid phase
SDE: Steam distillation and solvent extraction
DHS: Dynamic headspace sampling
SPME GC-O: Solid phase microextraction gas chromatography-olfactometry
GC-PFPD: Gas chromatography-pulsed flame photometric detector
HS-SPME: Headspace solid-phase micro-extraction
HS-SPME-GC-MS/MS: Headspace solidphase microextraction with gas chromatography-tandem mass spectrometry
HRGC/O: High-Resolution Gas Chromatography /Olfactometry
HRGC/MS: High-Resolution Gas Chromatography-Mass Spectrometry
HSSE/GC/MS: Headspace sorptive extraction Gas Chromatography-Mass Spectrometer
RI: Retention index
GC: Gas chromatograph
E-nose: Electronic nose
MOS: Metal oxide semiconductor
QDA: Quantitative descriptive analysis
NIRS: Near-infrared spectroscopy
HS: Headspace
GC-MS/MS: Gas chromatography-tandem mass spectrometry
SEM: Scanning electron microscopy
OSME: Olfactometry
GC-O: Gas chromatography-olfactometry
GC-O-MS: Gas Chromatography-Olfactometry-Mass Spectrometry
SDE: Steam distillation and solvent extraction
HS-GC-TOF MS: Headspace-gas chromatography-time-of-flight mass spectrometry
HS-SPME-GC-TOF MS: Headspace-solid phase microextraction-gas chromatography-time-of-flight mass spectrometry
APCR: Aseptic-packaged cooked rice
RGO: Graphene oxide
PCA: Principal Component Analysis
PNN: Probabilistic Neural Network
BPMLP: Back propagation multilayer perceptron
LDA: Linear Discriminant Analysis
FT-NIR: Fourier transformation near infra-red
OCT: Optimal cooking time
SCT: Suggested cooking time
CA: Clustering analysis
HPS: High pressure steam
CR: Cooling rates
